# Do Mathematics and Reading Skills Impact Student Science Outcomes?

**DOI:** 10.1177/00222194241263646

**Published:** 2024-07-26

**Authors:** Christian T. Doabler, Megan Rojo, Jenna A. Gersib, Anna-Maria Fall, Maria A. Longhi, Gail E. Lovette, Greg Roberts, Jasmine Uy, Katharina Johnson, Shadi Ghafghazi, Jason B. Phelps, Sarah R. Powell, William J. Therrien

**Affiliations:** 1 The University of Texas at Austinat Austin, Austin, TX, USA; 2 University of North Texas, Denton, TX, USA; 3 University of Virginia, Charlottesville, VA, USA

**Keywords:** science instruction, STEM, explicit instruction, vocabulary instruction, differential response, core instruction, moderation analyses, learning disabilities

## Abstract

Establishing validated science programs for students with or at risk for learning disabilities requires testing treatment effects and exploring differential response patterns. This study explored whether students’ initial mathematics and reading skills influenced their treatment response to a whole-class, second-grade science program called Scientific Explorers (Sci2). The original Sci2 study employed a cluster randomized controlled design and included 294 U.S. students from 18 second-grade classrooms. Differential effects of the program by initial mathematics and reading skill levels were not observed for an interactive science assessment and a distal science outcome measure. However, based on initial reading skill levels, moderation results were found on a science vocabulary measure, suggesting the effects of Sci2 were greatest for students with higher initial reading skills. Similar results were found using initial mathematics skill levels as a predictor of differential response such that students with higher mathematics skills reaped stronger treatment effects on the vocabulary measure. Further, we found initial mathematics skills also influenced outcomes on the proximal science content assessment, where students with higher initial mathematics skills led to higher outcomes. Overall, findings suggest Sci2 produced robust effects for all students (*g* = 0.24–1.23), regardless of initial skill proficiencies. Implications for exploring differential response in science intervention research are discussed.

Science, Technology, Engineering, and Mathematics (STEM) workers are of growing importance to our nation’s global economy. A diverse cadre of skilled STEM workforce can lead to new products, groundbreaking discoveries, and innovative companies and industries. However, forecasts for the next decade suggest the demand for STEM workers will continue to significantly outpace that of their non-STEM counterparts (National Science Board, 2020). These workforce demands raise concerns over the supply of trained STEM workers and whether the U.S. educational system can adequately prepare a future STEM workforce (National Science & Technology Council, Committee on STEM Education, 2018). Consequently, these workforce concerns have produced an educational flashpoint for reimagining U.S. STEM education.

Because improving U.S. STEM education is a complicated enterprise, it will require efforts on multiple fronts. One initial step is to ensure that core science instruction is effectively designed and delivered in today’s science classrooms. Critically, core (Tier 1) science instruction plays a vital role in the development of science literacy among all learners, including students with or at risk for learning disabilities (LD) in reading and/or mathematics. Above all, it is tasked with delivering instruction that supports all students in meeting the grade-level performance expectations recognized in national and state science standards (e.g., Next Generation Science Standards [NGSS], NGSS Lead States, 2013). For example, in second grade, core science instruction should help students understand cause-and-effect relationships when investigating the effect of animals pollinating plants (Nordine & Lee, 2021).Encouragingly, randomized controlled trials (RCTs) have begun to establish a body of empirical literature on core elementary science programs, suggesting promising effects on student science achievement and other science-related outcomes. Llosa et al. (2016) conducted a cluster RCT involving *Promoting Science Among English Language Learners* (P-SELL), a fifth-grade curricular and professional development intervention focused on earth, life, and physical sciences. A total of 5,728 fifth-grade students from 66 classrooms participated in the study. Overall, findings favored treatment over comparison schools on a proximal measure of science achievement (*d* = 0.25) and a state science assessment (*d* = 0.15). In another cluster RCT, Gray et al. (2022) examined the effects of *Zoology One*, an integrated literacy and science intervention for kindergarten classrooms. Zoology One employs explicit instruction to directly teach targeted reading and science content. This study, which involved 1,600 kindergartners in 71 classrooms, reported significant treatment effects on students’ reading comprehension (∆= .16), letter naming (∆= .28), and motivation to read (∆= .32). However, group differences were not found on the researcher-developed science assessment. More recently, Krajcik et al. (2023) investigated the efficacy of the *Multiple Literacies in Project-Based Learning* (ML-PBL), a discovery-based, third-grade science intervention focused on improving students’ academic, social, and emotional learning. Employing a cluster RCT, Krajcik and colleagues worked with 2,371 third-grade students from 46 schools in Michigan. Treatment teachers implemented ML-PBL, while control employed business-as-usual science instruction. Results suggested significant treatment effects on student science achievement (*g* = 0.29). Finally, Harris et al. (2023) conducted a cluster RCT, randomly assigning 40 schools to implement either *Amplify Science* (treatment) or a comparison condition that delivered business-as-usual science instruction. The study administered (a) two science assessments, (b) a reading achievement measure, and (c) a researcher-developed, group-administered, science vocabulary measure that included 10 science terms aligned with the NGSS first-grade performance expectations. Significant findings were observed on the two science assessments, with effect sizes (Hedges’ *g*) ranging from 0.05 to 0.24. Harris et al. observed the largest treatment effects on the vocabulary measure (*g* = 0.46).

## Research on Differential Response in Academic Interventions

Although efficacy data produced from methodologically rigorous studies, such as Llosa et al. (2016), Gray et al. (2022), Krajcik et al. (2023), and Harris et al. (2023), are undoubtedly crucial for building the evidentiary basis of science programs, these results alone are insufficient for understanding why and for whom interventions work. Therefore, in addition to establishing the efficacy of academic interventions, researchers have long explored student-level characteristics to understand differential response to academic instruction. The earliest endeavors of such moderation work were called *aptitude-by-treatment interaction* (ATI) research (Cronbach & Snow, 1977). However, researchers have advocated for steering away from ATI terminology given its focus on unalterable variables (Yoder & Compton, 2004). Instead, over the past two decades, the field has reimagined moderation research to focus more on malleable factors that predict treatment response (D. Fuchs & Fuchs, 2019). This paradigm shift is beginning to infiltrate science intervention research and thus prioritize the needs of students with or at risk of LD.

The work of Connor et al. (2012) provides an example of alterable student characteristics and moderated treatment effects in the context of Tier 1 core science instruction. Utilizing direct observations of second- and third-grade classroom instruction, Connor and colleagues explored whether the initial vocabulary, reading, and content-area knowledge of 61 second- and third-grade students moderated associations between specific types of observed science activities and student gains in content-area literacy. Science activities were codified into two dimensions: *management* and *delivery*. The authors investigated the dimension of management to unpack whether the instruction was led by the teacher, co-led by the teacher and student, or child/peer managed. The dimension of delivery focused on explicit lessons, discussion and activities, and text reading, with the discussion and activities being considered less explicit and more interactive. A major finding of this work was that Connor et al. detected an interaction in the second-grade data between child/peer-managed activities and content knowledge (science) scores. For students with higher pre-intervention content knowledge scores (i.e., lower risk), more time was spent on activities independent of teacher support, and greater gains were found in content-area knowledge. Conversely, for students with lower initial content knowledge scores (i.e., at or below the 25th percentile), more time in independent activities (e.g., sustained silent reading activities) was associated with weaker content knowledge gains. These results align with recommendations from experts that some explicitness of instruction is necessary to build content knowledge among students at risk for academic failure (Zhang et al., 2022).

In 2017, Connor et al. investigated the efficacy of *Content-Area Literacy Instruction* (CALI), an individualized, kindergarten through fourth-grade science and social studies program that has an overall aim of improving students’ passage comprehension. The CALI program’s two science units integrate literacy and science instruction against a backdrop of the 5E inquiry-based instructional model (Bybee et al., 2006). A total of 418 kindergarten through fourth-grade students participated in the efficacy trial. Overall, CALI was found to increase student science knowledge, with a large effect (*d* = 2.1). Connor et al. also noted an interaction effect for the science units such that students with lower initial passage comprehension scores demonstrated greater science gains than peers with higher incoming scores.

More recently, Kim et al. (2021) employed a cluster RCT to text the efficacy of the *Model of Reading Engagement* (MORE) intervention on students’ science domain knowledge in 34 first-grade classrooms. The MORE intervention is a first-grade content literacy intervention comprised of 10 lessons situated in the context of building students’ conceptual understanding of Arctic animals and the impact of human interaction on their survival. Approximately 650 students participated in the efficacy trial and impact results suggested a significant, positive treatment effect on students’ understanding of a set of proximal science vocabulary terms directly taught in the program (Hedges’ *g* = 0.30). Kim et al. also found evidence of MORE’s impact on distal measures of listening comprehension (*g* = 0.40), argumentative writing (*g* = 0.24), and reading comprehension (*g* = 0.11). In addition, the study reported evidence of differential response to the MORE intervention by student’s initial reading comprehension levels, such that intervention effects were stronger for students with higher pre-treatment reading comprehension scores.

## Reading and Mathematics as Predictors of Response to Early Science Instruction

The research by Connor et al. (2012, 2017) and Kim et al. (2021) produced important evidence for understanding the role initial reading skills play in understanding response variation to science instruction in the elementary grades. In science education, reading skills are of central importance because they allow students to attain knowledge of core disciplinary ideas and crosscutting concepts. Proficient reading skills, such as reading comprehension, enable students to interpret expository science text and extract its salient information effectively. Moreover, early vocabulary knowledge can enable students to produce scientifically accurate written and oral responses during science instruction (National Research Council [NRC], 2012).

Interestingly, Connor et al. (2012, 2017) and Kim et al. (2021) also spotlighted a notable gap in the existing empirical literature. Currently, there is insufficient evidence to determine whether students’ initial skills in mathematics influence science outcomes for students with or at risk for LD. Like reading, initial skills in mathematics also play an important role in students’ development of scientific knowledge and the application of the practices associated with science and engineering (NRC, 2012). In recent years, expectations around developing and using mathematics skills in science have significantly increased. This can be mainly attributed to the advent of the NGSS (NGSS Lead States, 2013), which require students to use and call upon their mathematics and computational thinking to demonstrate their understanding of science. For example, mathematically proficient students can interpret data and access information communicated through multiple modalities, including tables and figures. Moreover, they can accurately use mathematical vocabulary when communicating their mathematical thinking and problem-solving. However, for students with or at risk for LD in mathematics, acquiring science proficiency can be challenging. Difficulties faced with multi-step word problems, for example, can be a barrier for solving such problems in the context of science instruction. Thus, we contend that exploring malleable, student-level academic variables, such as initial reading and mathematics skill levels, that associate with varied STEM treatment outcomes could help the field eliminate disparities in STEM education and thus best meet the instructional needs of a wide range of learners, including students with or at risk for LD. If, for example, researchers find compelling evidence for poor response to a science program among second-grade students with low initial mathematics skills, it could spark course corrections to the program such as embedding more instructional scaffolding when students learn how to represent continuous science-related data on tables and graphs. The same holds for reading. Low response could warrant greater support for students to hear, say, and use key science vocabulary. Thus, this study explored whether response to a second-grade science program differed according to students’ reading and mathematics skills at pretest.

## Purpose of the Study

Arguably, core science instruction is essential for student science learning. For many students, including students with or at risk for LD, core science instruction provided in general education classrooms represents their sole source of science instruction and learning (Morningstar et al., 2017; U.S. Department of Education, 2022; Vannest et al., 2009). Relative to reading and mathematics, scant supplemental supports are available for students who struggle in science. Therefore, core science instruction holds responsibility for promoting science literacy for all students, including students who experience learning difficulties.

Against that backdrop, this study explored whether and to what extent Scientific Explorers (Sci2), a whole-class second-grade science program (Doabler, Therrien, et al., 2021), was differentially effective for students based on their initial skills in mathematics and reading. Doabler, Therrien, et al. (2021) investigated the initial efficacy of the Sci2 program to promote students’ knowledge of core disciplinary ideas, crosscutting concepts, and the science and engineering practiced related to Earth’s Systems in the NGSS (NGSS Lead States, 2013). Specifically, the Sci2 program focuses on how the natural processes of wind and water can rapidly and slowly change Earth’s landscape. To meet the needs of all students, including students with or at risk for LD, the Sci2 program embraces a systematic, explicit instructional approach. In this way, the program offers explicit directions so teachers can directly teach key science vocabulary and provide guided support to students during the scientific investigations.

This study uniquely examined how students’ initial mathematics and reading skills influence their response to science education. Understanding if students with low and high initial academic skills benefit equally from whole-class science instruction is crucial for adapting teaching methods. For example, if there is a differential response such that students with low initial reading skills do not reap equal benefit, additional support or instruction may be needed to ensure these at-risk learners can access and deeply engage in core science instruction.

To test the promise of the Sci2 program, Doabler, Therrien, et al. (2021) randomly assigned 18 second-grade classrooms within schools to treatment or comparison conditions. Teachers in treatment classrooms (*n* = 9) implemented the Sci2 program, whereas comparison classrooms (*n* = 9) utilized a combination of district-developed science materials and lessons from STEMscopes, a commercially available science program. The study included 294 second-grade students (141 treatment and 150 comparison). Overall, the findings suggested promise of the Sci2 program. Of the four science outcome measures administered in the initial efficacy trial, results indicated the Sci2’s program had a significant impact on two distal, research-developed science measures and a proximal science vocabulary assessment, with effect sizes (Hedges’ *g*) ranging from 0.48 to 0.94. A nonsignificant effect (*g* = 0.02) was found on a commercially available, distal science outcome assessment.

Recently, Rojo et al. (2024) utilized data from the Sci2 efficacy trial (Doabler,Therrien, et al., 2021) to explore whether there was evidence of differential response to the Sci2 program based on a set of student socio-demographic variables, including race, ethnicity, disability status, socioeconomic status, and gender. These moderation analyses focused on the four science outcome measures targeted in Doabler, Therrien, et al. (2021). Rojo et al. (2024) found that students from diverse backgrounds benefited equitably from the Sci2 program as demonstrated on the proximal science vocabulary measure and the two researcher-developed distal science assessments. While exploratory, these findings suggest that implementing purposefully-designed science programs, like Sci2, into early science education can foster inclusive learning environments, which is crucial for students from groups traditionally underrepresented in STEM (e.g., students with disabilities). Furthermore, Rojo et al. (2024) highlighted the importance of integrating validated instructional design and delivery principles in early STEM programs, such as building essential background knowledge, explicitly teaching academic vocabulary, providing scaffolded learning opportunities, offering timely feedback, and facilitating meaningful discussions, to mitigate socio-demographic opportunity gaps.

While the exploratory work of Rojo et al. (2024) was cogent, it did not examine whether students’ initial reading and mathematics skill levels influenced the program’s treatment effects. As noted, knowledge and application of reading and mathematics are fundamental to accessing critical science content and low performance in these content areas suggests the likelihood of facing learning difficulties in science (NRC, 2012). Therefore, the current study analyzed data from Doabler, Therrien, et al. (2021) to explore how students’ initial mathematics and reading skills influenced their response to the Sci2 program. Understanding if students with low and high initial academic skills benefit equally from whole-class science instruction is crucial for adapting teaching methods. For example, if there is a differential response such that students with low initial reading skills do not reap equal benefit, additional support or instruction may be needed to ensure these at-risk learners can access and deeply engage in core science instruction.

Two aims guided the current exploration study. First, given Sci2’s systematic, explicit instructional approach, we sought to observe whether the program would benefit the full range of learners, including students with or at risk for LD. Decades of high-quality, methodological research has crystallized the beneficial impact of systematic, explicit instruction on important academic outcomes for students with or at risk for LD (e.g., L. S. Fuchs et al., 2021; Vaughn et al., 2022). Therefore, experts posit that the positive effects of this approach can translate to science instruction for students with or at risk for LD (Therrien et al., 2011; Zhang et al., 2022). In this study, we operationalize a student’s risk status for LD as a pre-treatment score one standard deviation or greater below the mean on a standardized, middle-of-year mathematics or reading outcome measure. We rely on this definition because research suggests that low academic performance in mathematics and reading at the beginning and mid-year of elementary school is highly predictive of students failing to meet end-of-year grade level expectations and beyond (Morgan et al., 2009, 2011). Consequently, such performance difficulties may indicate further difficulties and possible identification of LD in subsequent grade levels. A second aim was to explore whether addressing the needs of students with or at risk for LD would come at the expense of the science achievement of typical and higher-performing students. We contend that well-designed and delivered core science instruction should promote science literacy for the full range of learners. The following two research questions were explored:

**Research Question 1 (RQ1):** Did students benefit differentially from Sci2 by initial early mathematics skills?
**Research Question 2 (RQ2):** Did students benefit differentially from Sci2 by initial early reading skills?

## Method

The initial efficacy of Sci2 was examined in a cluster RCT during the 2019–2020 school year (Doabler, Therrien, et al., 2021). The trial included 18 second-grade classrooms in a large public school district in central Texas. The classrooms were blocked by school and randomly assigned to receive the Sci2 program or business-as-usual science instruction. This study explored student science outcome data collected in the original efficacy trial (Doabler, Therrien, et al., 2021).

### Participants

#### Students

The school district’s demographic data reported a student population that was 9% Black or African American, 31% Hispanic, 38% White, 18% Asian, 4% Two or More Races, less than 1% Native American, and less than 1% Pacific Islander. In addition, 28% of the students were eligible for free or reduced-priced lunch, 11% were Emergent bilingual/English learners (EB/ELs), and 15% received special education services. Parental consent was sought for all students in the 18 participating second-grade classrooms. Of the 346 parental consents distributed, 294 (85%) consents were obtained. The analytic sample included a total of 294 students. Table 1 provides the available demographic information for participating students by condition along with the percentage of students in the full sample who tested at or below the 25th percentile on the mid-year aimswebPlus (https://app.aimswebplus.com/) reading and mathematics outcome measures. The 25th percentile is a cut point where students are conventionally considered at risk for LD (Fletcher et al., 2019). The sample included in this study mirror national trends in risk status and represents a full range of learners, including those with or at risk of LD based on aimswebPlus normative data (National Computer Systems [NCS] Pearson, Inc., 2017).

**Table 1.** Demographics for Student Participants in the Second-Grade Scientific Explorers Program Study.

Variables	Sci2		Comparison		Total	
	*n*	%	*n*	%	*n*	%
Gender						
Female	64	44.4	70	46.7	134	45.6
Male	77	53.5	80	53.3	157	53.4
Ethnicity						
Asian	34	23.6	43	28.7	77	26.2
African American	17	11.8	15	10	32	10.9
Hispanic or Latino	33	22.9	33	22	66	22.4
Two or More Races	7	4.9	3	2	10	3.4
White	50	34.7	56	37.3	106	36.1
Student eligible for special ed.	19	13.2	24	16	43	14.6
English learners	5	3.5	8	5.3	13	4.4
Eligible for free/reduced lunch	32	22.2	28	18.7	60	20.4
aimswebPlus scores						
≤25 percentile NSF	26	19.4	26	17.3	52	18.1
≤25 percentile CA	18	13.0	23	15.3	31	14.2
≤25 percentile ORF	32	23.4	26	17.3	58	20.2
≤25 percentile RC	22	15.9	15	10.0	37	16.3
≤25 percentile VOC	16	11.6	14	9.3	30	10.4

*Note*. Sci2 = second-grade Scientific Explorers program; NSF = number sense fluency and represents a composite number sense outcome; CA = concepts and applications, a mathematics outcome; ORF = oral reading fluency; RC = reading comprehension; VOC = vocabulary, a reading outcome.

#### Teachers

A total of 18 certified teachers from three schools participated in the study. The teachers identified as female and White. In addition, two of the teachers identified as Hispanic or Latino. The teachers had an average of 10.92 years of teaching experience, and four of the 18 teachers had a Master’s degree. The teachers delivered science instruction in English. One of the treatment classrooms was considered a dual-language classroom and therefore provided science instruction in Spanish before the study. The students in the dual-language program were proficient in English and were participating in the dual-language program to receive bilingual instruction. The district permitted the students to receive science instruction in English while participating in the study.

### Treatment Condition

Treatment teachers implemented the Sci2 program in whole class settings, 3 to 5 days per week. The Sci2 program includes 10 lessons (30–45 min each) focused on earth science. When designing Sci2, we adhered to the recommendations of the NGSS (NGSS Lead States, 2013) by promoting a “three-dimensional approach” to science learning. This approach helps students to explore and understand the world around them by making direct connections among the three dimensions of science: disciplinary core ideas, science and engineering practices, and crosscutting concepts (NRC, 2012; Nordine & Lee, 2021).

Sci2 fosters three-dimensional science learning through systematic, explicit design and delivery principles (Kame’enui & Simmons, 1999). This instructional architecture offers purposefully sequenced instructional examples to promote knowledge building, and frequent opportunities for teachers to model demonstrate and explain key science topics. Sci2 further supports students’ three-dimensional science learning by integrating validated early literacy activities (i.e., direct vocabulary routines and shared expository book reading) into science instruction. This integrated approach (Hwang et al., 2022) also facilitates science vocabulary development and opportunities for students to engage in science-related discourse.

Lessons in the Sci2 program include five instructional activities. The first activity, *Spark Your Thinking*, reviews prior content, activates students’ background knowledge, and builds student interest in the lesson’s targeted phenomenon. The second activity, *Vocabulary*, pre-teaches a select set of key science vocabulary terms. In these activities, vocabulary terms are introduced in student-friendly definitions. Students then have opportunities to (a) observe pictures of the terms in relevant scientific contexts and (b) hear examples of the terms in sentences. Next, with a partner, students discuss what they know about the new vocabulary terms and then share with the group at large. *Read-Aloud* is the program’s third activity. It contains an interactive reading activity where a teacher uses researcher-developed expository texts to foster whole-class discourse opportunities around new science concepts, vocabulary, and practices. The books include captivating characters (e.g., Gina the Geologist) that reoccur throughout the Sci2 program. Such characters are intended to pique students’ interest in science and help them envision themselves as scientists. The Read Aloud activity also allows students to contemplate guiding questions about the targeted phenomenon they will explore in the collaborative investigation activities.

The fourth activity, *Investigation*, allows students to (a) develop models to observe and investigate patterns in the natural world, (b) design solutions to slow or prevent movement of water from changing the shape of the land’s surface, (c) communicate findings through scientific discourse, and (d) critique and evaluate alternative explanations (NRC, 2012). The program’s scientific investigations utilize a mix of physical and simulation-based models to provide varied opportunities for students to “act as scientists” when interacting with natural phenomena, such as weathering, erosion, and deposition. Each investigation is approximately 20-minutes in duration and can extend across consecutive days of instruction. For example, the program contains a multi-day investigation about rainfall and how it can change natural landscapes. At the start of the investigation, students are asked about a phenomenon such as, “How does the amount of rain affect the erosion on a mountainside?” Students then interact with a simulation-based model of the mountain and a thunderstorm and make predictions about the plausible cause and effects of the storm on the mountainside. Next, students use their technology-based tablets to make observations of the storm’s effects on the mountain’s soil and rocks. Students then document the results in their journals and examine their predictions.

Each lesson concludes with a final activity called *Share Your Thinking*. These wrap-up activities serve as a way for the entire class to collaboratively “put the pieces together” and discuss takeaways from the day’s lesson. To engage students in scientific discourse, each Share Your Thinking activity centers on a whole-class discussion of evidence and findings generated in the lesson’s collaborative investigation. The program also includes a weekly home component allowing students to share what they learned during the week with their families.

In this study, treatment teachers implemented the Sci2 program across approximately 2 to 3 weeks. The average number of students per treatment classroom was 17.1 (*SD* = 3.2). Across the study, Sci2 lessons averaged 41.2 min (*SD* = 10.1). All treatment teachers participated in two 3-hour professional development workshops before the onset of the Sci2 program. In each workshop, teachers received opportunities to teach portions of the Sci2 lessons and receive feedback on the quality of lesson implementation from research staff. Treatment teachers also received three to four coaching visits from Sci2 curriculum development team members. Such visits focused on providing feedback to teachers on the quality of student-teacher interactions and adherence to the Sci2 program.

### Comparison Condition

Comparison classrooms implemented business-as-usual science instruction that comprised commercially available (STEMscopes; Accelerate Learning, 2023) and district-developed science materials. STEMscopes is a core science program that employs an inquiry-based approach, prioritizes science content identified in the NGSS (NGSS Lead States, 2013), and includes hands-on labs, simulated experiences, and science reading. The duration of each science lesson in control classrooms was, on average, 37.8 min (*SD* = 7.6). Comparison classrooms had an average of 18.1 (*SD* = 1.4) students.

To document science instruction in the comparison classrooms, trained research staff conducted two real-time observations per classroom. Observation data indicated that the science instruction provided in comparison classrooms focused on topics related to Earth’s systems and was delivered in a whole-class setting. Educational technology was also an observed instructional medium in the comparison classrooms. However, observations noted comparison students had no direct interactions with educational technology. Instead, teachers used technology to display science content on large screens during whole-class instruction.

Some comparison classrooms were observed using hands-on investigations and engaging in read-alouds of narrative and expository science books. Among the teachers who included vocabulary instruction in their science core lessons, the vast majority facilitated implicit vocabulary instructional techniques. In addition, observation data revealed that 33% of observed lessons in comparison classrooms integrated concepts of mathematics, and 67% incorporated written response opportunities. Finally, while most of the science instruction in comparison classrooms focused on natural phenomena, such as the weathering of rocks, few teachers clarified the connections between science content and lesson activities.

### Fidelity of Implementation

Trained research staff conducted fidelity checks in all treatment and comparison classrooms. Each classroom was observed twice, and we found no evidence of treatment diffusion. Research staff used a 12-item observation protocol to measure implementation fidelity in treatment classrooms. Ten of the items employed a three-point rating scale. On these items, observers rated the extent to which treatment teachers implemented: (a) lesson scripts (*M* = 2.61, *SD* = 0.50); (b) simulation-based activities (*M* = 3.00, *SD* = 0.00); (c) activities as designed (*M* = 2.67, *SD* = 0.50); (d) Sci2 instructional formats (*M* = 2.83, *SD* = 0.38); (e) Spark Your Thinking (*M* = 3.00, *SD* = 0.00); (f) *Vocabulary* (*M* = 2.67, *SD* = 0.82); (g) *Vocabulary Extension* (*M* = 2.67, *SD* = 0.71); (h) *Read Aloud* (*M* = 2.67, *SD* = 0.82); (i) *Investigation* (*M* = 2.50, *SD* = 0.52); and (j) *Share Your Thinking* (*M* = 1.40, *SD* = 0.90). The final two items applied a five-point scale (1 = low, 3 = medium, 5 = high) to capture observers’ overall impressions of treatment teachers’ fidelity of implementation and the quality of student–teacher interactions during Sci2 instruction. Findings suggested treatment teachers implemented the Sci2 program with strong fidelity (*M* = 4.06, *SD* = 0.73). The quality of student–teacher interactions around targeted science content was considered moderate (*M* = 3.78, *SD* = 0.81).

### Science Outcome Measures

We used four science assessments, each administered at pretest and posttest, to determine the effects of Sci2 on student understanding and application of the second-grade NGSS core disciplinary ideas, crosscutting concepts, and science and engineering practices (NGSS Lead States, 2013). In addition, we harvested district-administered assessment data from the winter benchmark administration of three mathematics subtests and three reading subtests from aimswebPlus (NCS Pearson, Inc., 2017). This study used these data as predictors of students’ differential response to the Sci2 program.

#### Measure of Science Vocabulary Knowledge (SEVA)

The Measure of Science Vocabulary Knowledge (SEVA) measures students’ ability to orally define 10 science terms recognized in second-grade national and state science standards. Each item is worth two points (i.e., 20 possible points), with one point for partially correct responses and zero points for incorrect responses. To administer the SEVA, the assessor asks individual students to define each word (e.g., ”What does the word *erosion* mean?”). The 10-item SEVA fit the model very well, supporting the items’ unidimensional structure, χ^2^ (35) = 65.01, *p* < .001, SRMR = .06, TLI = .96, and CFI = .97. The information function converted to the traditional measure of reliability was above .90 in the average range of the trait continuum (from ~ 0.50*SD* below the mean to 1.5 above the mean). Outside of this range, reliability was .80. Cronbach’s alpha for the sample was .76, and test–retest reliability was .65.

#### Virtual Interactive Scientific Practices Assessment

The Virtual Interactive Scientific Practices Assessment (VISPA; Doabler, Longhi, Uy, et al., 2019) is an individually administered, distal assessment that measures students’ application and understanding of the science and engineering practices identified in the NGSS (NGSS Lead States, 2013). Students interact with a virtual character for this assessment to plan and conduct a scientific investigation focused on wind erosion. The VISPA is administered through a tablet and contains six items. Each item is worth two points for 12 possible points, with partially correct items receiving one point and incorrect responses resulting in zero points. The six-item VISPA fit the model very well, supporting the items’ unidimensional structure, χ^2^(9) = 9.72, *p* = .374, SRMR = .04, TLI = .97, and CFI = .98. Item Response Theory (IRT)-based test information function converted to a traditional measure of reliability (reliability = information/[information + 1]) indicated that reliability was above .60 for ability levels ranging from −2 to 2. The reliability coefficient for Cronbach’s alpha was .53 and for test–retest reliability was .44.

#### Test of Early Geoscience Learning

The Test of Early Geoscience Learning (TEGL; Doabler, Longhi, Maddox, et al., 2019) measures students’ understanding of Earth processes (e.g., weathering and erosion) identified in the second-grade science standards. The TEGL is a group-administered, distal assessment containing 16 multiple-choice, one check all that apply, and three short answer items. The multiple-choice items are each worth one point, check all that apply is worth three points, and the short answer questions are each worth two points, for a total of 25 possible points. The 20-item TEGL fit the model well, χ^2^ (170) = 240.66, *p* < .001, SRMR = .08, TLI = .91, and CFI = .92. The selected items measured the targeted constructs with the greatest information available in the average ability distribution (θ). IRT-based test reliability was above .70 for ability levels ranging from −2.80 to 0.60. Cronbach’s alpha was .71 and test–retest reliability was .63.

#### Content Knowledge and Scientific Practices

The Content Knowledge and Scientific Practices (CKSP; Assessment Technology Inc [ATI], 2019) is a distal outcome measure that purports to assess students’ knowledge of the core disciplinary ideas and crosscutting concepts related to Earth’s systems in the second-grade NGSS (NGSS Lead States, 2013). This group-administered assessment comprises 10 multiple-choice items, each worth one point. Reported Item Response Theory includes three parameters: discrimination (0.82 to 1.41), difficulty (−1.58 to 2.42), and guessing (0.23 to 0.40; ATI, 2019). The 10-item CKSP fit the model very well, supporting the items’ unidimensional structure, χ^2^ (35) = 45.75, *p* = .11, SRMR = .08, TLI = .93, and CFI = .95. The information function converted to the traditional measure of reliability was above .60 in the negative range of the trait continuum (from ~3*SD* below the mean to −1.5*SD* below the mean), after which point reliability drops to .40. Cronbach’s alpha was .41 and test–retest reliability was .55.

### Measures of Differential Response

#### aimswebPlus

The aimswebPlus system (NCS Pearson, Inc., 2017) is a computer-based assessment designed for screening and progress monitoring reading and mathematics skills in kindergarten through eighth grade. In reading, participating students were assessed on Oral Reading Fluency (ORF), Vocabulary (VOC), and Reading Comprehension (RC). Except ORF, all reading measures are untimed, multiple-choice, and group administered. The ORF measure is an individually administered, fluency-based (1-min) measure that assesses students’ skill in accurately and fluently reading connected text. The mathematics assessments are multiple-choice and administered in group settings. In mathematics, participating students were assessed on Number Comparison Fluency (NCF-T), Mental Computation Fluency (MCF), as well as Concepts and Applications (CA). Internal consistency for the reading measures is also moderate to strong (α = .67 to .87) and concurrent validity correlations with statewide reading achievement data range from .77 to .85 (Klingbeil et al., 2022). Technical adequacy of aimswebPlus indicate the mathematics measures have moderate to strong internal consistency (α = .77–.85) and mean concurrent validity coefficients range from .68 to .80 (Klingbeil et al., 2022). Participating schools administered the aimswebPlus measures as the second-grade winter reading and mathematics benchmarks. Administration time occurred within 2 weeks from the beginning of the original Sci2 efficacy trial. In this study, we aggregated scores based on the aimswebPlus guidelines (NCS Pearson, Inc., 2017) from the three reading assessments to serve as a marker of students’ pre-treatment reading performance and, thus a potential predictor of differential response. To form an overall reading composite, we calculated the simple mean of the ORF, VOC, and RC scores. For the three mathematics assessments, we calculated the sum across the NCF-T and MCF scores to create an average Number Sense Fluency (NSF) score and then added the NSF score with CA to generate an overall average mathematics composite score.

### Data Analysis

The statistical models described in this paper extend earlier work reported by Doabler, Therrien, et al. (2021), which focused on the main effects of the Sci2 program. Here, we address variables that may moderate the efficacy of the Sci2 program (Preacher et al., 2016). We modeled two pre-treatment academic performance factors as potential moderators: reading and mathematics initial skills. For reading and mathematics, we estimated the moderating effect of one while controlling for pretest performance on the other, which we label “control variable” in equations (1) and (2).

Students were nested within classrooms and classrooms were nested within schools. To explore the effects of nesting, we initially fit three-level unconditional models for all four outcome variables of interest. Initial unconditional models indicated that there was no school-related variation for any of the four measures. Thus, we removed the random effects at the school levels and estimated a more parsimonious two-level model with students nested in classrooms. For TEGL and CKSP, these two-level models resulted in singular fit, indicating that the models are overfitted and random effects at the classroom level was not supported by the data. Thus, we removed the random effects and estimated a single-level model for TEGL and CKSP.

Predictors in the moderation models (Preacher et al., 2016) for TEGL and CKSP included assignment (*Sci2_i_*), the grand mean centered pretest score for the outcome (*Pretest_i_*), the grand mean centered control variable (*Control variable_i_*), the grand mean centered moderator (*Moderator_i_*), and the interaction of assignment and the moderator (Sci2*Moderator)_i_:

(1) $$\begin{array}{c} Y_{i}=\;\beta_{0}+\;\beta_{1}{\left(Pretest\right)}_{i}+\;\beta_{2}{\left(SCI2\right)}_{i}\\ +\;\;\beta_{3}{\left(Control\;variable\right)}_{i}+\;\beta_{4}{\left(Moderator\right)}_{i}\;\\ +\;\beta_{5}{\left(SCI2*Moderator\right)}_{i}\;+\;e_{i}. \end{array}$$

For VISPA and SEVA, moderation analysis was examined using multilevel moderation in Equation 2:

(2) $$\begin{array}{c} Y_{ij}\;=\;Y_{00}\;+\;Y_{10}\;{\left(Pretest\right)}_{ij}\;+\;Y_{01}{\left(Pretest\right)}_{j}\;\\ +\;Y_{20}\;{\left(Control\;variable\right)}_{ij}\;+\;Y_{02}{\left(Control\;variable\right)}_{j}\;\\ +\;Y_{30}\;{\left(Moderator\right)}_{ij}\;+\;Y_{03}{\left(Moderator\right)}_{j}\;+\;Y_{04}SCI2_{j}\;\\ +\;Y_{21}\;{\left(SCI2*Moderator\right)}_{ij}\;+\;Y_{22}{\left(SCI2*Moderator\right)}_{j}\\ +\;e_{ij}+\;\;u_{0j}, \end{array}$$

where *Y_ij_* was the student-level posttest score, *Moderator_ij_*, *Control variable_ij_*, and *Pretest_ij_* were student-level variables centered around classroom mean, *Moderator_j_, Control variable_j_, and Pretest_.j_* were classroom-level variables centered around the grand mean. *Sci2_j_* was classroom-level intervention variable, γ_21_ represented the cross-level interactions between moderator (student-level) and treatment assignment (classroom-level). γ_03_ described the interaction of Sci2 and the between-groups part of the moderator. We estimated the between and within components of the moderator and pretest covariate to decompose the effect across different levels of the factor and allow for unbiased estimates of student- and class-level effects without conflating level-specific effects (Preacher et al., 2016).

We ran the analyses using R’s *lme4* package (Bates et al., 2015). We followed up significant interactions using the Johnson–Neyman (J-N) technique (Preacher et al., 2006), which produces values along the full range of the moderator at which the effect of Sci2 transitions from statistically significant to non-significant. These values demarcate the regions of significance for the intervention effect (Lin, 2020). We also calculated effect sizes (*g*) for the outcomes at low (1*SD* below the sample mean), average (at the sample mean), and high (1*SD* above the sample mean) levels of the moderator by dividing the differences between adjusted means (i.e., treatment mean − control mean) by the pooled standard deviation for the outcome measure.

Given the exploratory nature of the study, we elected not to correct for Type 1 error associated with multiple comparisons. In studies with limited sample sizes and diminished levels of statistical power, the application of stringent correction methods can be counterproductive (Rothman, 1990; Saville, 1990).

## Results

A detailed description of the Sci2 program’s main effects is reported in Doabler, Therrien, et al. (2021). Students in classrooms assigned to the Sci2 program significantly outperformed students in control classrooms on three of the four outcomes, including SEVA, a proximal science vocabulary measure (*g* = 0.48); VISPA, a distal interactive assessment focused on the NGSS science and engineering practices (*g* = 0.94), and TEGL, a distal multiple-choice measure focused on Earth’s system (*g* = 0.60). Doabler, Therrien, et al. (2021) also reported positive, albeit nonsignificant, effects on the CKSP, a distal science outcome measure (*g* = 0.02).

### Initial Mathematics Skills

**Figure 1.**
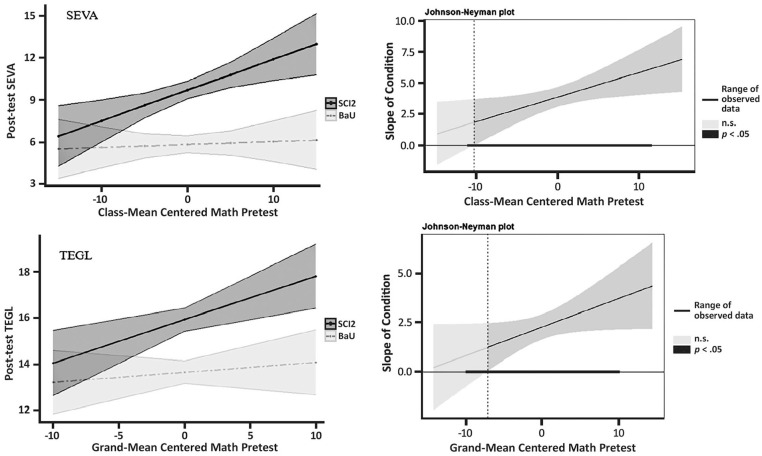
Moderation Effects of Initial Mathematics Scores on SEVA and TEGL. *Note.* Visualization of mathematics as a moderator of intervention effect on SEVA (top left) and TEGL (bottom left) score at posttest (predicted mean + 95% *CI* on the left). Confidence bands and regions of significance for each moderator are presented in gray. The dotted line marks the score of the moderator at which the association between Sci2 and mathematics becomes statistically significant (*p* < .05). The range of the moderator is presented using a solid bold line. SEVA = Measure of Science Vocabulary Knowledge; TEGL = Test of Early Geoscience Learning (Doabler, Longhi, Maddox, et al., 2019).

On average, the composite score for the aimswebPlus mathematics assessment was 203.09 (*SD* = 44.14). The individual measures, including NCF-T (*M* = 10.04, *SD* = 9.70), MCF (*M* = 16.10, *SD* = 10.75), and CA (*M* = 21.39, *SD* = 6.31) represented a range of initial skill levels across the participating students. Of interest, 14.2% (NSF) and 18.1% (CA) of students in the full sample scored at or below the 25th percentile based on aimswebPlus normative data (see Table 1; NCS Pearson, Inc., 2017) . Full results representing the moderating effects of initial mathematic performance are reported in Table 2. Mathematics skill levels did not moderate the efficacy of the Sci2 program on the VISPA and CKSP outcomes. However, pretest performance in mathematics moderated scores on the SEVA and the TEGL. The significant effects indicated that as students’ pretest mathematic scores increased, a greater effect of the Sci2 program on the SEVA was observed (see Figure 1). A follow-up Johnson–Neyman plot suggested that the effect favoring the Sci2 program over control remained significant for all students (*n* = 293), including those with or at risk for LD.

**Table 2.** Initial Skills in Reading and Mathematics as Moderators of Differences in Posttest Scores.

Measure	Fixed effects	Mathematics			Reading		
		Estimate	*SE*	*p*-value	Estimate	*SE*	*p*-value
SEVA	Intercept	5.81	0.28	.00	5.80	0.27	.00
	Pretest L1	0.65	0.07	.00	0.67	0.07	.00
	Pretest L2	0.67	0.28	.04	0.69	0.28	.03
	Sci2	3.86	0.41	.00	3.87	0.40	.00
	Control variable L1	0.18	0.04	.00	0.11	0.06	.06
	Control variable L2	0.07	0.15	.67	0.35	0.19	.09
	Moderator L1	0.02	0.07	.76	0.12	0.05	.02
	Moderator L2	0.28	0.22	.22	–0.01	0.16	.97
	Sci2 × Moderator L1	**0.20**	**0.08**	**.02**	**0.13**	**0.06**	**.03**
	Sci2 × Moderator L2	0.20	0.18	.28	0.18	0.12	.15
VISPA	Intercept	6.75	0.21	.00	6.76	0.21	.00
	Pretest L1	0.37	0.06	.00	0.37	0.06	.00
	Pretest L2	0.54	0.33	.13	0.44	0.33	.21
	Sci2	1.08	0.31	.01	1.04	0.32	.01
	Control variable L1	0.00	0.03	.92	0.13	0.04	.00
	Control variable L2	–0.07	0.12	.57	0.20	0.15	.19
	Moderator L1	0.10	0.05	.05	0.01	0.04	.86
	Moderator L2	0.26	0.16	.13	–0.05	0.12	.69
	Sci2 × Moderator L1	0.06	0.06	.34	–0.02	0.04	.71
	Sci2 × Moderator L2	–0.11	0.14	.43	–0.01	0.10	.94
TEGL	Intercept	13.61	0.23	.00	13.62	0.23	.00
	Pretest	0.42	0.06	.00	0.42	0.06	.00
	Sci2	2.28	0.33	.00	2.28	0.33	.00
	Control variable	0.24	0.04	.00	0.11	0.05	.05
	Moderator	0.04	0.07	.51	0.22	0.05	.00
	Sci2 × Moderator	**0.14**	**0.07**	**.05**	0.06	0.05	.28
CKSP	Intercept	6.00	0.11	.00	6.00	0.11	.00
	Pretest	0.36	0.06	.00	0.36	0.06	.00
	Sci2	0.00	0.16	.99	0.00	0.16	.99
	Control variable	0.04	0.02	.02	0.08	0.03	.01
	Moderator	0.08	0.03	.02	0.05	0.02	.02
	Sci2 × Moderator	0.00	0.04	.92	–0.02	0.03	.41
Measure	Random effects	Variance	ICC		Variance	ICC	
SEVA	Level 1	7.12	0.97		7.14	0.98	
	Level 2	0.21	0.03		0.17	0.02	
VISPA	Level 1	4.24	0.98		4.25	0.98	
	Level 2	0.09	0.02		0.11	0.02	

*Note.* Bold text indicates statistically significant effect (*p*-value < 0.05). SEVA = Measure of Science Vocabulary Knowledge; VESPA = Virtual Interactive Scientific Practices Assessment (Doabler, Longhi, Uy, et al., 2019); TEGL = Test of Early Geoscience Learning (Doabler, Longhi, Maddox, et al., 2019); CKSP = Content Knowledge and Scientific Practices (Assessment Technology Inc, 2019); Sci2 = second-grade Scientific Explorers program; L1 = Level 1, L2 = Level 2. ICC = interclass correlation coefficient.

Table 3 displays the effect sizes for the SEVA based on students’ initial mathematics skills relative to the sample population (i.e., 2*SD* below the mean, 1*SD* below the mean, at the mean, 1*SD* above the mean, and 2*SD* above the mean). Lower mathematics scores (i.e., at least 1*SD* below the sample mean, *n* = 52) were associated with a smaller impact on the Sci2 program (*g* = 0.52 to 0.70), but higher initial mathematics scores (i.e., at least 1*SD* above the sample mean, *n* = 147) were associated with a larger impact (*g* = 1.05 to 1.23) of the Sci2 program.

**Table 3.** Effect Sizes for Each Outcome at Different Levels of the Moderator.

ES for levels of moderator						
Measure	Moderator	−2 *SD* Math *n* = 3 Reading *n* = 14	−1 *SD* Math *n* = 49 Reading *n* = 32	*M* Math *n* = 89 Reading *n* = 74	+1 *SD* Math *n* = 91 Reading *n* = 131	+2 *SD* Math *n* = 56 Reading *n* = 36
SEVA	Math L1	0.52**	0.70***	0.87***	1.05***	1.23***
	Reading L1	0.56**	0.72***	0.88***	1.04***	1.19***
VISPA	Math L1	0.25	0.35*	0.45**	0.55**	0.67**
	Reading L1	0.51**	0.47**	0.44**	0.40**	0.36
TEGL	Math	0.24	0.39***	0.55***	0.70***	0.85***
	Reading	0.38*	0.46***	0.55***	0.63***	0.72***
CKSP	Math	0.02	0.01	0.00	–0.01	–0.02
	Reading	0.15	0.07	0.00	–0.08	–0.15

*Note*. SEVA = Measure of Science Vocabulary Knowledge; VESPA = Virtual Interactive Scientific Practices Assessment (Doabler, Longhi, Uy, et al., 2019); TEGL = Test of Early Geoscience Learning (Doabler, Longhi, Maddox, et al., 2019); CKSP = Content Knowledge and Scientific Practices (Assessment Technology Inc, 2019); L1 = Level 1.

* *p* < .05. **p < .01. ***p < .001.

On the TEGL, the effect of the Sci2 program was evident for all students regardless of initial mathematics skill level. The coefficient for the interaction was positive and statistically significant, indicating that the treatment effect favoring Sci2 becomes significantly larger for each additional point increase in mathematics (see Figure 1). Lower mathematics scores (i.e., students at least 1*SD* below the sample mean) were associated with a significant, yet smaller impact of the Sci2 program (*g* = 0.24 to 0.39), but higher mathematics scores (i.e., at least 1 *SD* above the sample mean) were associated with a larger impact (*g* = 0.70 to 0.85) of Sci2.

### Initial Reading Skills

**Figure 2.**
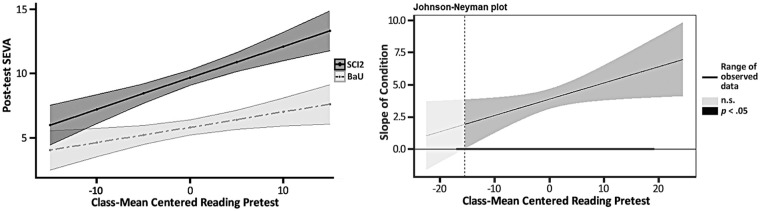
Moderation Effects of Initial Reading Scores on SEVA. *Note.* Visualization of reading as a moderator of intervention effect on SEVA score at posttest (predicted mean + 95% *CI* on the left). Confidence bands and regions of significance for each moderator are presented in gray. The dotted line marks the score of the moderator at which the association between Sci2 and mathematics becomes statistically significant (*p* < .05). The range of the moderator is presented using a solid bold line. SEVA = Measure of Science Vocabulary Knowledge; Sci2 = second-grade Scientific Explorers program.

For reading, the average composite score across all learners was 391.58 (*SD* = 63.67). The reading assessment measured VO (*M* = 11.69, *SD* = 2.82), ORF (*M* = 95.21, *SD* = 41.67), and RC (*M* = 16.37, *SD* = 5.67), where students’ initial reading scores spanned the spectrum of population-normed scores (see Table 3). In our sample, 10.4% (Vocabulary) to 20.2% (Oral Reading Fluency) of students fell at or below the normed aimswebPlus 25th percentile for determining LD risk status (NCS Pearson, Inc., 2017). Our findings suggest that initial skill levels did not moderate the efficacy of the Sci2 program on the VISPA, CKSP, or TEGL. Results for the moderating effect of initial reading performance at pretest are reported in Table 2. Moderating effects of initial reading skills were found on the SEVA, a science vocabulary measure. The positive effects indicated that as students’ pretest reading scores increased, a greater effect of the Sci2 program was observed (see Figure 2). A follow-up Johnson–Neyman plot suggested that the effect favoring Sci2 over control remained significant for all students. Effect sizes for the SEVA at relatively low, average, and high levels of reading performance based on sample population scores are shown in Table 3. Lower reading scores (i.e., at least 1*SD* below the sample mean) were associated with a smaller impact of the Sci2 program on the SEVA (*g* = 0.56 to 0.72), but higher reading scores (i.e., at least 1*SD* above the sample mean) were associated with a larger treatment effect (*g* = 1.04 to 1.19).

## Discussion

This study explored response variation to the Sci2 program based on students’ initial mathematics and reading skill levels. We had two aims of exploration. First, we explored whether there was differential response to the Sci2 program based on students initial skills in mathematics and reading. Second, we examined whether the Sci2 program would be efficacious for all students, including those students with or at risk for LD. Sci2 is built on a foundation of systematic, explicit instruction and the preponderance of evidence is clear and compelling in favor of using these validated design and delivery principles when working with students with or at risk for LD. Based on district provided data, between 10% and 20% of the current student sample tested below the 25th percentile on mid-year, reading and mathematics outcome measures. We were also interested in whether addressing the needs of students with or at risk for LD would not suppress the science outcomes of students with higher pre-treatment reading and mathematics skills. As noted, the goal of core instruction is to work for all students. In the following sections, we discuss the interpretation of our findings and their implications for research and practice.

This study contributes significantly to the literature by examining how initial mathematics and reading skills influence students’ response to core science instruction. Through moderation analyses, the study advances the field’s understanding of which students benefit most from science instruction, underscoring the importance of considering initial skill levels that students bring to the science classroom. Findings from studies like the current one, where a differential response was observed, can inform the need for targeted support and instruction to ensure all students can excel in science education and reach their potential in this critical domain.

### Initial Skills in Mathematics

First, we explored whether the treatment effects of Sci2 were moderated by students’ initial mathematics skills with whole numbers and operations. The exploration of pre-intervention mathematics skill levels as predictors of differential response remains in its infancy in science education. Therefore, this study represents one of the first to explore whether students differentially benefited from a Tier 1 science program by initial early mathematics skill. Results suggested effects of the Sci2 program were moderated by students’ mathematics skill levels such that students with higher initial skills demonstrated stronger performance on the TEGL, a distal, researcher-developed science assessment, and the SEVA, a measure of science vocabulary. One plausible explanation for these moderation effects is that students with higher initial mathematics skills can more easily transfer or generalize their mathematical thinking to science learning environments than peers with lower initial whole number skills.

Regarding differential effects between the SEVA and initial mathematics skills, our results indicate a linkage between mathematics skills and vocabulary knowledge. These findings align with prior research that suggests students use language as a medium to communicate, represent, and retrieve mathematics knowledge and facilitate working memory and reasoning during mathematics performance and learning (Peng et al., 2020). Research further suggests that students with or at risk for LD require frequent opportunities to verbalize their mathematical thinking and understanding, and receive performance feedback on such discourse opportunities (Doabler, Clarke, et al., 2021). As such, it is important that elementary classrooms foster verbalization opportunities for students with or at risk for LD within the context of science instruction, answering inferential questions such as “how” and “why.”

### Initial Skills in Reading

Next, we explored whether students’ initial reading skill levels moderated the effects of the Sci2 program. The results of this analysis indicated that students’ initial skills in reading comprehension, vocabulary, and reading fluency impacted the extent to which they profited from Sci2 as measured by the SEVA, our science vocabulary measure. Specifically, students with higher initial reading skills demonstrated greater science vocabulary outcome increases due to the Sci2 program. While our findings differed from those reported by Connor et al. (2017), they largely aligned with those found in Connor et al. (2012) and Kim et al. (2021) in that students with higher incoming reading skills appear to reap greater benefit from science instruction than students with lower initial reading skills.

The Matthew Effect, which suggests that individuals with a higher knowledge base can acquire new information faster (Stanovich, 1986), may explain this study’s findings. The Matthew Effect has been commonly studied around vocabulary acquisition (e.g., Coyne et al., 2019), demonstrating that students with higher vocabulary can leverage their existing knowledge to acquire new vocabulary at a faster rate than students with a lower vocabulary, thus widening the achievement gap in the early grades. In this study, it is plausible that students with higher pre-treatment reading skills were better able to evoke and capitalize on their prior reading knowledge and skill, particularly in Sci2’s vocabulary and read aloud activities. Regardless of the reason behind these findings, we may need to seek additional ways to better meet the early literacy needs of students with or at risk for LD. For example, we recommend that teachers offer multiple opportunities for students with or at risk for LD to use critical science vocabulary across other content areas, such as reading and mathematics. To illustrate, teachers could introduce the vocabulary words, *weathering* and *erosion*, during science instruction and extend the use of these terms to reading comprehension and history by reading a book related to the Dust Bowl. Later while teaching word problem-solving in mathematics, teachers could include the two terms in a comparison word problem, such as one that involves calculating the difference of soil depth before and after a windstorm.

### Overall Student Responsiveness in Terms of Treatment Effects

When exploring student responsiveness, we found the Sci2 program produced robust effects for all participating students in our sample on three outcome measures (*g* = 0.24 to 1.23). These findings suggest there was universal improvement for all students, regardless of initial skill levels in reading and mathematics. We credit Sci2’s effect robustness and capability to meet a wide range of student academic needs, including those of students with or at risk for LD, to the program’s systematic and explicit instructional design and delivery features. These features support teachers in facilitating guided and independent student practice opportunities. Sci2 applies this approach because the field has accumulated strong evidence suggesting that systematically designed and explicitly delivered instruction produces improved outcomes for at-risk learners (L. S. Fuchs et al., 2021; Vaughn et al., 2022).

### Limitations

Specific limitations should be noted. First, the school district administered the measures that were used to estimate students’ initial reading and mathematics skill levels (i.e., aimswebPlus). Therefore our research team did not monitor the fidelity of the assessment procedures. However, using extant data provided by school districts has important research-to-practice implications as it can help build partnerships with school communities, conserve project resources, and ensure results are meaningful to educators. The second limitation is that Sci2 is a 10-lesson program. While the lessons can be implemented daily or across multiple weeks, the generalization of these results may be limited to science programs with similar dosage levels. Given the variety of topics covered in early science instruction, we find this dosage level to be in line with other science instruction research (e.g., Kim et al., 2021) and appropriate as Earth science represents only one fraction of the yearlong science curriculum suggested by national and state standards for second-grade students. Thus, teaching the concepts targeted in the Sci2 program beyond 10 lessons can be challenging to fit into the school calendar. While the Sci2 program was overall efficacious for participating students, it is possible that a stronger dosage, such as offering learning opportunities beyond the school day, may be required for some students with lower initial mathematics and reading skills. Finally, the teacher sample was small. Consequently, the results should be considered preliminary and exploratory. However, we believe this study demonstrates an initial step in exploring the black box of science programs.

### Implications for Research and Practice

Our study has several implications for the field. First, given reading and mathematics skills are requisite for student science success, we encourage researchers to use moderation analyses to explore the influence of incoming academic skills on science program outcomes. Surprisingly, few science intervention studies have investigated initial mathematics skills as a predictor of differential response. Theoretically, reading and mathematics skills are distinct yet interconnected components contributing to science learning (NRC, 2012). Acquiring a robust and lasting understanding of early science literacy necessitates the ability to read complex text and fluently apply numerical concepts and operations. It is reasonable to argue that scientific problems cannot be solved using single isolated skills. Instead, solving science-related problems requires the integration of reading and mathematics skills. Thus, to grasp foundational science ideas and concepts, early elementary students must possess strong initial skills in reading and in mathematics and bring these skills to bear when attempting to make sense of the world. Thus, it seems prudent to explore whether treatment effects vary by students’ initial reading and mathematics skill levels. Such moderation research could yield significant insights into improving science education and reducing opportunity gaps for student with or at risk for LD.

Second, the overall responsiveness rate found in the current treatment sample aligns with the calls by some for the use of systematic, explicit instruction in elementary science education (Klahr & Nigam, 2004; Zhang et al., 2022). It may be that science instruction needs to be more systematically designed and explicitly delivered to meet the needs of the full range of learners, especially students with or at risk for LD. However, given the exploratory nature of the current research, future work that permits causal explanations is needed to build the evidentiary basis behind the application of systematic, explicit instruction in science education.

Finally, the results of our exploration work add further evidence for integrating aspects of early literacy instruction with content-area instruction (Hwang et al., 2022). When designing the Sci2 program, we purposefully embedded direct vocabulary routines and expository text read-alouds given their distinct importance for supporting three-dimensional science learning among students with or at risk for LD. Take, for example, vocabulary development. Research highlights the importance of building students’ expressive and receptive language (Coyne et al., 2019). Moreover, recent evidence suggests vocabulary instruction is a strong complement to science education, particularly when the goal is to reduce socio-demographic disparities in science achievement (Morgan et al., 2024). The Sci2 program, therefore, offers frequent occasions for students to develop and use specialized vocabulary throughout its lesson activities. First, teachers systematically pre-teach several key vocabulary terms in each lesson, offering clear and student-friendly definitions for each term. The vocabulary activities then provide examples of these terms and use corresponding images to contextualize the words. Next, students receive structured opportunities to discuss vocabulary terms in shared reading activities with expository and narrative science texts. The program offers continued vocabulary practice, allowing students to use the words as they gain experience with the targeted content during the collaborative investigations. The program’s final activities permit students to express their understanding and engage in whole-class discussions.

## Conclusion

Although conducting rigorous efficacy trials is fundamental to establishing what works in instruction, another critical step in building a cache of evidence-based academic programs is exploring under what conditions and for which participants such programs work. Therefore, researchers are starting to explore pre-treatment student-level academic performance variables as mechanisms for understanding differential responses to validated reading and mathematics interventions. However, few studies to date have examined differential responses in science instruction. Unpacking the black box of science programs may provide important curricular information for best meeting the instructional needs of students who face learning difficulties in science. The current exploration research sought to serve as an initial step in addressing this blank spot in the literature on early science instruction.
